# Phase and Valence State Engineering of MOFs-Derived Iron Oxide@Carbon Polyhedrons for Advanced Microwave Absorption

**DOI:** 10.3390/nano15110806

**Published:** 2025-05-27

**Authors:** Xiaojiao Yang, Shuai Han, Hongna Xing, Yi Dong, Xia Deng, Yan Zong, Juan Feng, Xiuhong Zhu, Xinghua Li, Xinliang Zheng

**Affiliations:** 1 State Key Laboratory of Photoelectric Technology and Functional Materials, School of Physics, Northwest University, Xi’an 710127, China; 202321672@stumail.nwu.edu.cn (X.Y.); zongy@nwu.edu.cn (Y.Z.); zhuxh@nwu.edu.cn (X.Z.); zhengxl@nwu.edu.cn (X.Z.); 2 College of Materials Science and Engineering, Hebei University of Engineering, Handan 056000, China; hanshuai@hebeu.edu.cn; 3 Electron Microscopy Centre, School of Materials and Energy, Lanzhou University, Lanzhou 730000, China; dongy2024@lzu.edu.cn (Y.D.); dengx@lzu.edu.cn (X.D.)

**Keywords:** phase transformation, iron oxide@C, mixed-valence, microwave absorption

## Abstract

MOFs-derived magnetic carbon-based composites are considered to be valuable materials for the design of high-performance microwave absorbents. Regulating phase structures and introducing mixed-valence states within the composites is a promising strategy to enhance their charge transfer properties, resulting in improved microwave absorption performance. In this study, iron oxide components show a temperature-dependent phase evolution process (α-Fe_2_O_3_→Fe_3_O_4_→Fe_3_O_4_/FeO), during which the valence states of iron ions are regulated. The tunable phases modulate the magnetic Fe_3_O_4_ component, resulting in enhanced magnetic loss. The changed valence states affect the polarization relaxation by adjusting the electronic structure and tune the electron scattering by introducing defects, leading to enhanced dielectric loss. The microwave absorption properties of iron oxide@carbon composites display phase- and valence state-dependent characteristics. Especially, Fe_3_O_4_@C composites exhibit superior microwave absorption properties, ascribed to the improved magnetic/dielectric losses induced by good impedance matching and strong microwave attenuation capacity. The minimum reflection loss of Fe_3_O_4_@C composites reaches −73.14 dB at 10.35 GHz with an effective absorption bandwidth of 4.9 GHz (7.69–12.59 GHz) when the absorber thickness is 2.31 mm. This work provides new insights into the adjustment of electromagnetic parameters and microwave absorption properties by regulating the phase and valence state.

## 1. Introduction

With the continuous progress of modern science and technology, electromagnetic technologies have become increasingly integrated into various aspects of contemporary life, such as communication, medical, military, and so on. The proliferation of electromagnetic devices has generated increasing electromagnetic pollution, which calls for the exploration of high-performance microwave absorbents [1,2,3,4,5,6]. Generally, ideal microwave absorbers are expected to simultaneously optimize four critical performance parameters: minimal thickness, reduced mass density, strong absorption capability and wide effective absorption bandwidth (EAB) [7,8,9,10]. Nevertheless, conventional microwave absorption materials face intrinsic limitations, for example, large density for ferrites and metals/alloys, easy oxidation for metals/alloys and impedance mismatching for carbon materials, which greatly restrict their performance and operational applicability [11,12,13,14,15,16,17]. Therefore, exploring novel microwave absorption materials is urgently demanded to meet the requirements of today’s complex and changeable electromagnetic environment.

Metal-organic frameworks (MOFs) are regarded as promising precursors for the construction of lightweight microwave absorption materials, owing to crystallographically tunable architectures, high porosity, large specific surface areas and excellent component processability [18,19,20,21]. These inherent characteristics enable precise modulation of electromagnetic parameters at molecular scales, particularly through strategic selection of metal nodes and organic linkers containing π-conjugated systems [22,23]. MOFs-derived magnetic carbon-based composites inherit these structural advantages of their MOF precursors, and simultaneously integrate dual loss mechanisms, with dielectric loss from the graphitized carbon matrix and magnetic loss from the embedded magnetic species. In addition, the inherited porous structure and high specific surface area is favorable for prolonging the transmission path of microwaves, enhancing the opportunity for interaction between microwaves and absorbents [24,25]. Magnetic species are homogeneously dispersed within the carbon matrix, thereby avoiding aggregation issues caused by magnetic attraction, leading to enhanced magnetic loss capability [26,27,28,29]. This synergistic configuration theoretically satisfies the essential requirements for high-performance microwave absorption, appropriate impedance matching and multi-scale energy conversion. Nowadays, the design of MOFs-derived magnetic carbon-based microwave absorption materials mainly focuses on the aspects of component modulation and multidimensional structural integration [30,31,32,33]. Novel perspectives are needed to deeply understand the enhanced microwave absorption mechanisms.

The strategic regulation of phase structures and introduction of mixed-valence states within composite materials is a promising strategy for the enhancement of interfacial charge transfer kinetics, thereby significantly improving dielectric dissipation capacity through intensified polarization relaxation processes [34,35,36,37]. These modulation mechanisms synergistically optimize impedance-matching conditions and amplify microwave attenuation capacity, ultimately achieving superior microwave absorption. To implement this paradigm, Fe-based MOF precursors were subjected to controlled annealing in Ar/H_2_ atmospheres at different temperatures, resulting in the formation of iron oxide@carbon composites with precise phase control of iron oxide species (Fe_2_O_3_/Fe_3_O_4_/FeO) and quantitative modulation of Fe^2+^/Fe^3+^ ratios. Through systematic investigation of phase composition evolution and valence state variations, this work establishes a definitive structure–performance correlation between the material’s crystallochemical characteristics (phase configuration, valence distribution) and its electromagnetic response mechanisms (ε″/μ″ variation, microwave absorption performance). This work bridges the gap between atomic-scale compositional engineering of MOF-derived magnetic carbon composites and macroscopic microwave absorption performance, providing new insights for developing novel microwave absorbents.

## 2. Materials and Methods

### 2.1. Materials

Iron trichloride hexahydrate (FeCl_3_·6H_2_O) was purchased from Sinopharm Chemical Reagent Co., Ltd (Shanghai, China). Terephthalic acid (PTA) was sourced from Aladdin (Shanghai, China). The chemicals were directly used as received.

### 2.2. Preparation of Fe-Based MOFs Precursor

First, 5 mmol of FeCl_3_·6H_2_O and 5 mmol of terephthalic acid were sequentially dissolved in 60 mL of deionized water under magnetic stirring at room temperature, forming a homogeneous solution. The resultant mixture was transferred into a 100 mL Teflon-lined stainless steel autoclave, which was then sealed and heated at 170 °C for 24 h. After natural cooling to room temperature, the reddish-brown precipitate was collected by centrifugation at 8000 rpm for 2 min, followed by alternating washing with deionized water and ethanol three times each. The purified product was dried at 60 °C for 24 h.

### 2.3. Preparation of Iron Oxide@Carbon Composites

Iron oxide@carbon composites were obtained by a carbonization process. In detail, an Fe-based MOF precursor was placed in a tubular furnace and heated under a flowing Ar/H_2_ gas mixture (90% Ar + 10% H_2_, 100 sccm). The heating rate and duration time were set as 2 °C·min^−1^ and 3 h, respectively. The carbonization temperatures were variously 350, 400, 450, 500, 550, and 600 °C, and the resulting samples were named as Fe_x_O_y_@C-T (T = 350, 400, 450, 500, 550, and 600).

### 2.4. Testing and Characterization

Microstructural analysis was conducted using a scanning electron microscope (SEM, Apreo S, ThermoFisher Scientific. Waltham, MA, USA) and a transmission electron microscope (TEM, Tecnai G2 F20, FEI. Hillsboro, OR, USA). The SEM operated at an accelerating voltage of 10 kV, and the TEM operated at an accelerating voltage of 300 kV. Phase structure was characterized via X-ray diffraction (XRD, D8 Advance, Bruker. Beijing, China) using Cu Kα radiation (λ = 1.5406 Å) operated at 40 kV and 30 mA. The diffraction patterns were collected in the range of 10° to 90°. Surface elemental composition and chemical states were studied by X-ray photoelectron spectroscopy (XPS, ESCALAB Xi^+^, ThermoFisher Scientific. Waltham, MA, USA) using an Al target. The electromagnetic parameters were evaluated using a vector network analyzer (VNA, Agilent Technologies N5232A. Santa Rosa, CA, USA) in the frequency range of 0.1–18 GHz with 801 sampling points and an intermediate frequency bandwidth (IFBW) of 300 Hz. The samples were uniformly mixed with paraffin wax with a filling ratio of 70 wt%, and then pressed into toroidal-shaped specimens (outer diameter: 7.0 mm, inner diameter: 3.04 mm). Prior to measurement, a full two-port SOLT (Short-Open-Load-Thru) calibration was performed using standard calibration kits. The complex permittivity (ε) and permeability (μ) were derived from S-parameters via the Nicholson–Ross–Weir (NRW) algorithm using the co-axial transmission/reflection method.

## 3. Results and Discussion

**Figure 1:**
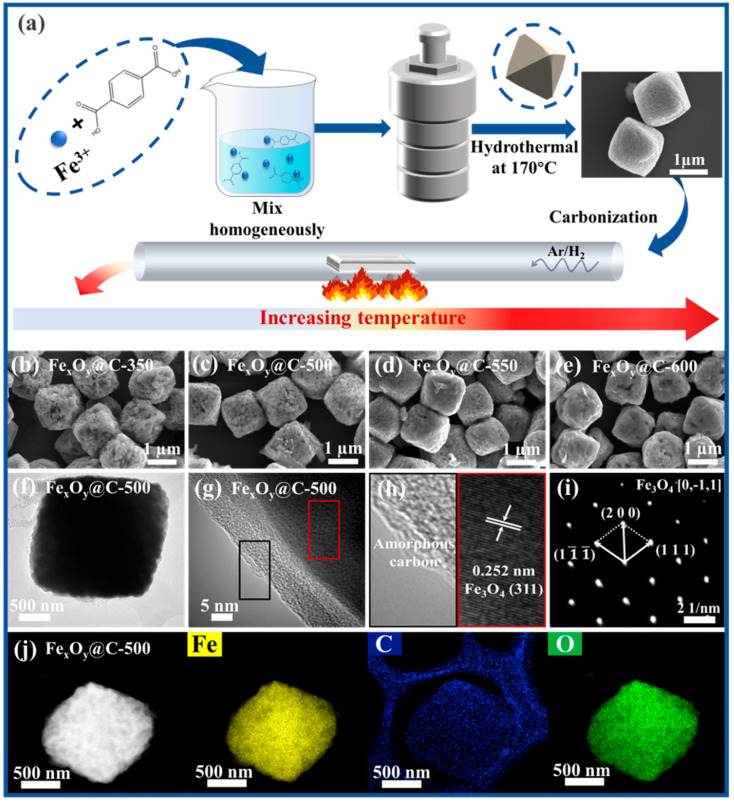
(**a**) Illustration of the preparation process of the Fe-based MOFs precursor and derivatives. (**b**–**e**) SEM images of Fe_x_O_y_@C-T. (**f**) TEM, (**g**,**h**) HRTEM and (**i**) SAED images of Fe_x_O_y_@C-500. (**j**) HAADF-STEM image and corresponding EDX elemental mappings of a single Fe_x_O_y_@C-500 particle.

Figure 1a illustrates the preparation process of the Fe-based MOFs precursor and the derived composites. The Fe-based MOFs precursor was first synthesized by a hydrothermal route, using Fe^3+^ and terephthalic acid as the metal node and organic linker, respectively. The Fe-based MOFs show a micro-scale polyhedral shape with porous structure. Fe_x_O_y_@C-T derivatives were fabricated by carbonization treatment of Fe-based MOFs in Ar/H_2_ atmosphere, during which the phases of iron oxide were modulated by simply changing the annealing temperatures. SEM images of Fe_x_O_y_@C-T composites (Figure 1b–e) reveal that the samples retained the well-defined polyhedral morphology of the parent Fe-based MOFs, with individual Fe_x_O_y_@C particles measuring ~1.5 μm in size. By increasing the carbonization temperature, the porous structure of Fe_x_O_y_@C-T derivatives progressively collapses, resulting in a concomitant increase in surface smoothness. Representative TEM image (Figure 1f) of a single Fe_x_O_y_@C-500 particle shows sharp-edged polyhedral morphology, accordant with the SEM results. HRTEM images (Figure 1g,h) show that the Fe_x_O_y_ particles are coated by a thin amorphous carbon layer. The interplanar spacing of Fe_x_O_y_@C-500 is measured to be 0.252 nm, corresponding to the (311) crystal plane of cubic Fe_3_O_4_. A SAED image of a single Fe_x_O_y_@C-500 particle (Figure 1i) displays monocrystalline characteristic. Figure 1j presents a HAADF-STEM image along with the corresponding EDX elemental mappings of a single Fe_x_O_y_@C-500 particle. Fe, C, and O elements are uniformly distributed throughout the particle region, suggesting that Fe_x_O_y_ is uniformly embedded within the carbon framework (the detected carbon signal around the particles is caused by the carbon film).

**Figure 2:**
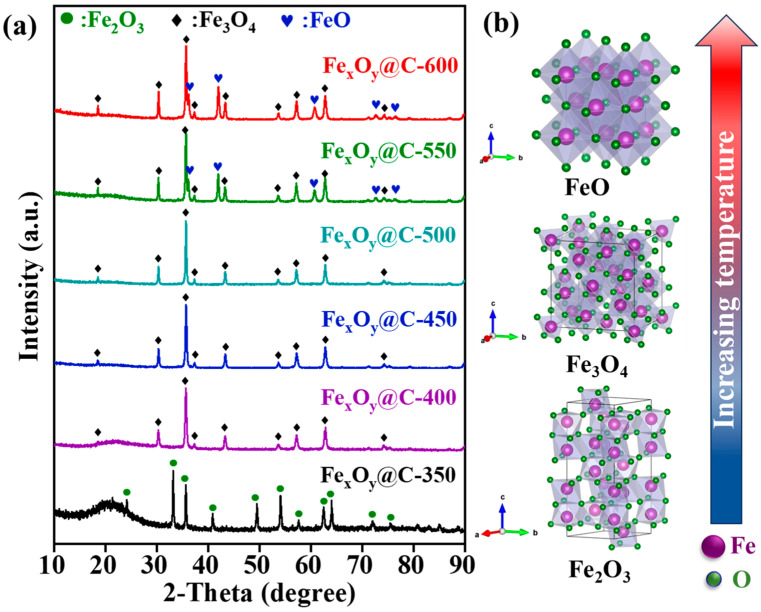
(**a**) XRD patterns and (**b**) crystallographic phase evolution of Fe_x_O_y_@C-T.

The crystallographic phase evolution of Fe_x_O_y_@C-T composites under controlled carbonization temperatures was systematically elucidated through XRD analysis (Figure 2), demonstrating precise phase engineering from antiferromagnetic hematite (α-Fe_2_O_3_) to ferrimagnetic magnetite (Fe_3_O_4_) and ultimately paramagnetism wüstite (FeO). Initial carbonization at 350 °C exclusively yielded hexagonal α-Fe_2_O_3_ with a pace group of R-3c (No. 167), identified by characteristic diffraction peaks at 24.2°, 33.2°, 35.6°, 40.8°, 49.5°, 54.1°, 57.6°, 62.5°, 64.0°, 72.3°and 75.5° which correspond to the (012), (104), (110), (113), (024), (116), (018), (214), (300), (119) and (220) crystal planes of α-Fe_2_O_3_, respectively (JCPDS No. 79-1741). Elevating temperatures to 400–500 °C triggered complete phase transformation from hexagonal α-Fe_2_O_3_ into cubic spinel Fe_3_O_4_ with an Fd-3m space group, demonstrated by the disappearance of all characteristic peaks of α-Fe_2_O_3_ and the emergence of diffraction peaks at 18.3°, 30.2°, 35.5°, 37.2°, 43.2°, 53.6°, 57.1°, 62.7° and 74.2°, corresponding to the (111), (220), (311), (222), (400), (422), (511), (440) and (533) crystal planes of Fe_3_O_4_ (JCPDS No. 88-0315). Progressive annealing at 550–600 °C induced phase conversion from Fe_3_O_4_ to FeO, evidenced by the emergence of diffraction peaks at 35.9°, 41.7°, 60.5°, 72.4° and 76.2°, attributed to the (111), (200), (220), (311) and (222) crystal planes of FeO (JCPDS No. 89-0687). This transformation is attributed to the hydrogen-induced reduction reaction through oxygen vacancy formation: Fe_3_O_4_ + H_2_ → 3FeO + H_2_O.

**Figure 3:**
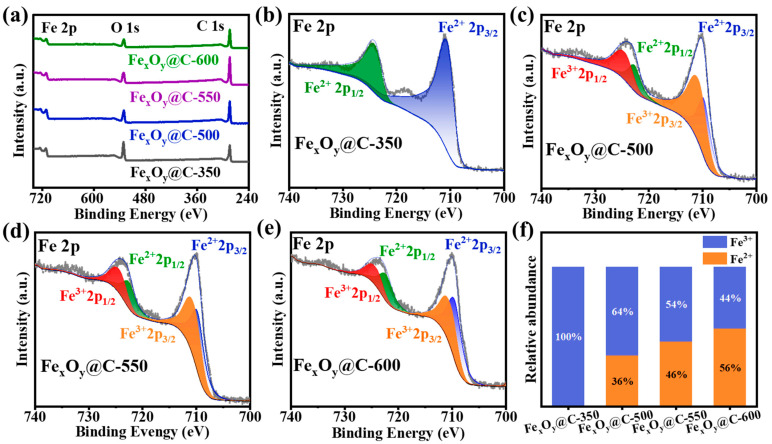
(**a**) Full scan XPS spectra and (**b**–**e**) high-resolution Fe 2p spectra of Fe_x_O_y_@C-T. (**f**) Quantitative analysis of the Fe^3+^/Fe^2+^ proportions in Fe_x_O_y_@C-T.

XPS analysis of Fe_x_O_y_@C-T composites (Figure 3) was employed to systematically elucidate the temperature-dependent evolution of iron oxidation states, complementing XRD phase characterization. The survey spectra (Figure 3a) confirm the presence of C, O, and Fe elements with distinct C 1s (284.8 eV), O 1s (531.5 eV), and Fe 2p (710–725 eV) signals, consistent with the previous SEM and XRD observations. High-resolution Fe 2p spectra (Figure 3b–e) show the characteristic spin-orbit doublets at ~710 eV for Fe 2p_3/2_ and ~725 eV for Fe 2p_1/2_, with satellite peaks at ~719 eV and ~733 eV for iron oxides. Fe 2p_3/2_ can be fitted into two peaks with 711.5 eV for Fe^3+^ and 710 eV for Fe^2+^. Quantitative deconvolution analysis of peak areas (Figure 3f) reveals temperature-dependent valence state evolution: progressive Fe^3+^ content is reduced from 100% (Fe_x_O_y_@C-350) to 44% (Fe_x_O_y_@C-600). This valence evolution aligns with the phase transformations observed by XRD. Fe_x_O_y_@C-350 contains solely Fe^3+^ ions, attributed to its pure α-Fe_2_O_3_ phase. Fe_x_O_y_@C-500 reveals a Fe^2+^/Fe^3+^ ratio of about 1:2, consistent with its Fe_3_O_4_ (FeO·Fe_2_O_3_) phase. Further temperature elevation induced progressive reduction of Fe^3+^ to Fe^2+^, with Fe_x_O_y_@C-550 and Fe_x_O_y_@C-600 showing increasing Fe^2+^ proportions as partial Fe_3_O_4_ was transformed into FeO via hydrogen-mediated reduction. The complementary XRD and XPS characterizations conclusively validate the phase evolution (Fe_2_O_3_ → Fe_3_O_4_ → Fe_3_O_4_/FeO) and progressive Fe^2+^/Fe^3+^ valence state modulation.

The Fe_x_O_y_@C-T composites were uniformly dispersed in paraffin wax at a filler loading of 70 wt%, followed by compression into a toroidal shape for electromagnetic characterization. Given that pure paraffin wax is transparent to microwaves, it is commonly used as a binder in microwave absorption materials to hold fillers together. The electromagnetic parameters of microwave absorption materials serve as critical indicators for characterizing the interaction with electromagnetic waves, encompassing loss mechanisms and impedance matching characteristics. Microwave absorption performance fundamentally depends on the material’s complex permittivity ($\varepsilon_{r}=\varepsilon^{\prime }-j\varepsilon^{\prime \prime }$) and complex permeability ($\mu_{r}=\mu^{\prime }-j\mu^{\prime \prime }$), where the real components ($\varepsilon^{\prime }$, $\mu^{\prime }$) quantify energy storage capacity, while the imaginary components ($\varepsilon^{\prime \prime }$, $\mu^{\prime \prime }$) reflect energy dissipation efficiency [17].

**Figure 4:**
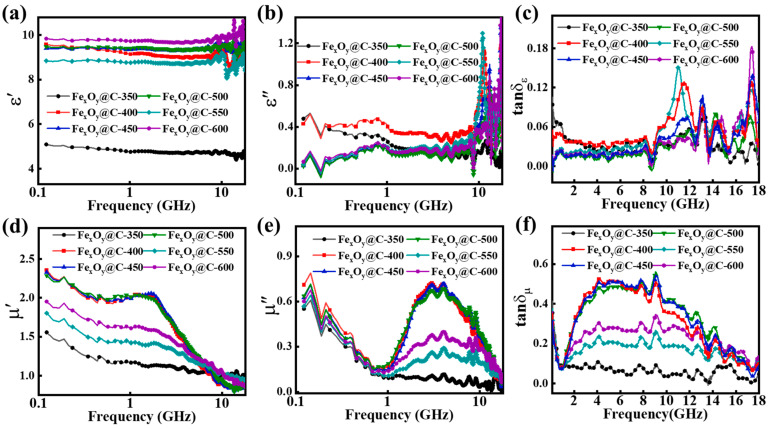
Temperature-modulated electromagnetic parameters of Fe_x_O_y_@C-T composites: (**a**) $\varepsilon^{\prime }$, (**b**) $\varepsilon^{\prime \prime }$, (**c**) $\tan\delta_{\varepsilon }$, (**d**) $\mu^{\prime }$, (**e**) $\mu^{\prime \prime }$ and (**f**) $\tan\delta_{\varepsilon }$.

Figure 4a,b presents the complex permittivity of the Fe_x_O_y_@C-T composites. Contrary to conventional observations that elevated carbonization temperatures typically improve the graphitization degree of carbon materials and raise the electrical conductivity and complex permittivity [38,39,40], these Fe_x_O_y_@C-T composites exhibit a non-monotonic permittivity evolution. In Fe_x_O_y_@C-350 composites, the minimal permittivity is ascribed to the combined effects of a weak graphitization degree of the carbon matrix caused by low carbonization temperature and limited electron transport in the single-phase Fe_2_O_3_ with sole Fe^3+^. For Fe_x_O_y_@C-400/450/500 composites, the phase transition to Fe_3_O_4_ introduces Fe^2+^ ions, establishing a Fe^2+^/Fe^3+^ mixed-valence system within the spinel structure. This configuration enables electron hopping between the adjacent Fe^2+^ and Fe^3+^ in spinel structure, which would accelerate the electron transition process and induce dipole polarization relaxation within crystal lattices, resulting in enhanced complex permittivity and dielectric loss. Notably, the comparable complex permittivity values observed between Fe_3_O_4_@C-450 and Fe_3_O_4_@C-500 composites reveal a carbonization temperature-insensitive dielectric response, suggesting that the carbon matrix’s graphitization degree exerts minimal influence on complex permittivity. Compared with the Fe_3_O_4_@C system, Fe_x_O_y_@C-550 exhibits diminished complex permittivity, primarily attributed to weakened Fe^2+^/Fe^3+^ charge transfer caused by the progressive FeO phase formation. In contrast, Fe_x_O_y_@C-600 demonstrates significantly enhanced permittivity due to two synergistic effects: (1) The formation of well-defined Fe_3_O_4_/FeO heterojunctions through phase evolution facilitates intensified interfacial polarization at grain boundaries; (2) the hydrogen reduction-induced oxygen vacancies during valence state transformation create localized defect dipoles that amplify polarization relaxation. These dual mechanisms cooperatively optimize electromagnetic energy conversion through enhanced polarization loss pathways.

Figure 4d,e delineates the complex permeability of Fe_x_O_y_@C-T composites, suggesting non-monotonic $\mu^{\prime }$ (*f*) and $\mu^{\prime \prime }$ (*f*) variations modulated by phase transition. The variation tendency of complex permeability directly correlates with the phase transition sequence: antiferromagnetic Fe_2_O_3_ → ferrimagnetic Fe_3_O_4_ → ferrimagnetic Fe_3_O_4_/paramagnetic FeO, as governed by carbonization temperature. The improved permeability in Fe_x_O_y_@C-400/450/500 composites is ascribed to emergent ferrimagnetic Fe_3_O_4_. The subsequent paramagnetic FeO formation disrupts long-range magnetic ordering, resulting in reduced values for Fe_x_O_y_@C-550/600 composites. Notably, the $\mu^{\prime \prime }$ curves of the Fe_3_O_4_@C system display a broad resonance spanning range of 1–10 GHz, corresponding to the natural ferromagnetic resonance of ferrimagnetic Fe_3_O_4_. The broad resonance bandwidth is associated with the distribution of domain sizes and variations of crystalline anisotropy energy.

The dielectric loss tangent ($\tan\delta_{\varepsilon }=\varepsilon^{\prime \prime }/\varepsilon^{\prime }$) and magnetic loss tangent ($\tan\delta_{\mu }=\mu^{\prime \prime }/\mu^{\prime }$) serve as quantitative metrics for evaluating electromagnetic energy dissipation characteristics, where $\tan\delta_{\varepsilon }$ quantifies dielectric loss and $\tan\delta_{\mu }$ corresponds to magnetic loss [41]. As demonstrated in Figure 4c,f, both parameters exhibit distinctive frequency-dependent behaviors. Notably, the evolution of $\tan\delta_{\mu }$ values shows strong correlation with crystallographic phase evolution, where increased concentration of ferrimagnetic Fe_3_O_4_ significantly boosts the magnetic loss. Comparative analysis of loss tangents reveals $\tan\delta_{\mu }$ > $\tan\delta_{\varepsilon }$ for Fe_x_O_y_@C-400/450/500 composites, establishing magnetic loss as the predominant energy dissipation mechanism. This dominance can be attributed to natural resonance characteristics arising from the ferrimagnetic Fe_3_O_4_ phase formation.

**Figure 5:**
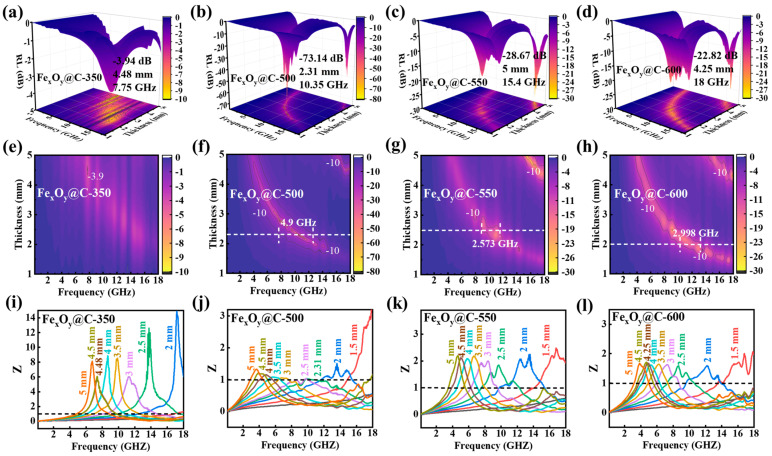
(**a**–**d**) 3D *RL* representations, (**e**–**h**) 2D *RL* contour profiles and (**i**–**l**) Z curves of Fe_x_O_y_@C-T composites (The white dashed lines in (**f**–**h**) represent the optimal absorber thicknesses with the broadest EAB. The black dashed lines in (**i**–**l**) represent Z = 1).

The reflection loss (*RL*) serves as the principal metric for evaluating microwave absorption performance, derived from transmission line theory through the following equations [17]:

(1) $$RL=20\log\left|\frac{Z_{in}-Z_{0}}{Z_{in}+Z_{0}}\right|$$

(2) $$Z_{0}=\sqrt{\mu_{0}/\varepsilon_{0}}$$

(3) $$Z_{in}=Z_{0}{\left(\mu_{r}|\varepsilon_{r}\right)}^{1/2}\tanh\left\{j\left(2\pi fd/c\right){\left(\mu_{r}\varepsilon_{r}\right)}^{1/2}\right\}$$

where $Z_{0}$ (377 Ω) denotes the intrinsic impedance of free space, $Z_{in}$ is the input impedance of absorber, *f* is the microwave frequency, *d* is the absorber thickness, and *c* is the velocity of light. The *RL* values directly reflect the microwave absorption efficiency, where lower *RL* values (more negative) correspond to reduced electromagnetic wave reflection and enhanced absorption capabilities. Quantitatively, an *RL* threshold of −10 dB corresponds to 90% wave attenuation, defining the effective absorption bandwidth (EAB) as the frequency range satisfying RL ≤ −10 dB, which serves as a critical parameter determining practical applicability [42]. Figure 5a–h presents the typical three-dimensional (3D) *RL* mappings and corresponding contour profiles of Fe_x_O_y_@C-T composites. The microwave absorption properties are significantly affected by the phase transition of iron oxide caused by carbonization temperatures. Fe_x_O_y_@C-350 (Fe_2_O_3_@C) shows weak absorption capabilities with *RL* > −10 dB in the entire frequency range, caused by the ultralow dielectric loss and negligible magnetic loss. Fe_x_O_y_@C-550 and Fe_x_O_y_@C-600 (Fe_3_O_4_/FeO@C) exhibit effective microwave absorption (*RL* ≤ −10 dB) in specific frequency bands through optimal thickness modulation. However, both of them exhibit relatively low absorption capabilities, narrow EAB and greater absorber thickness: Fe_x_O_y_@C-550 reveals a minimum *RL* (*RL*_min_) value of −28.7 dB at 5.0 mm, while the EAB is only 2.6 GHz at 2.5 mm; the *RL*_min_ value of Fe_x_O_y_@C-600 is only −22.8 dB at 4.25 mm, accompanied by an EAB of 3 GHz at 2.0 mm. Notably, Fe_x_O_y_@C-500 (Fe_3_O_4_@C) displays an *RL*_min_ value of −73.14 dB at 10.35 GHz and a broad EAB of 4.9 GHz (7.69–12.59 GHz) with a thin thickness of only 2.31 mm. Table 1 summarizes the comparison of microwave absorption properties of MOFs-derived Fe_3_O_4_@C with other reported Fe_3_O_4_-based composites [42,43,44,45,46,47,48]. The Fe_3_O_4_@C composites prepared in this work display remarkable *RL*_min_ value and moderated EAB at a relatively thin thickness. The superior microwave absorption properties of Fe_x_O_y_@C-500 primarily stem from the synergistic interplay between improved magnetic loss and optimized dielectric loss characteristics. This performance enhancement is achieved through precisely engineered phase composition and valence state manipulation, which facilitates broadband ferromagnetic resonance for improved magnetic loss while simultaneously establishing optimal interfacial polarization and dipole polarization effects for balanced dielectric loss. Additionally, as shown in Figure 1b–e, Fe_x_O_y_@C-T composites exhibit abundant porous structures. The porous structure is beneficial because it introduces rich air–material interfaces, enhancing multiple reflections and scattering of incident electromagnetic waves. This is due to the effect described by the Maxwell–Garnett (MG) effective medium theory [49]:

(4) $$\varepsilon_{eff}^{MG}=\varepsilon_{1}\frac{\left(\varepsilon_{2}+2\varepsilon_{1}\right)+2p\left(\varepsilon_{2}-\varepsilon_{1}\right)}{\left(\varepsilon_{2}+2\varepsilon_{1}\right)-p\left(\varepsilon_{2}-\varepsilon_{1}\right)}$$

where $\varepsilon_{1}$ and $\varepsilon_{2}$ represent the permittivity of solid component and air component, respectively, and p denotes the volume ratio of air. The porous structure can effectively reduce the equivalent permittivity of composites, thereby optimizing impedance matching.

The enhanced microwave absorption capabilities of microwave absorbers arise from dual fundamental mechanisms: optimized impedance matching characteristic and efficient electromagnetic attenuation capacity [50]. Impedance matching, quantified by the normalized impedance parameter ($Z=\left|Z_{in}/Z_{0}\right|$), determines the electromagnetic wave penetration efficiency through minimal interfacial reflection. Optimal absorption occurs when Z approaches unity (Z → 1), indicating complete transmission of incident electromagnetic waves into the microwave absorber. Figure 5i–l presents the normalized impedance (Z) curves of Fe_x_O_y_@C-T composites by varying absorber thicknesses. Notably, Fe_x_O_y_@C-500 exhibits optimal impedance-matching characteristics with Z values close to 1. Electromagnetic attenuation capacity is mathematically described by the attenuation coefficient α, which combines both dielectric and magnetic loss components through the following relationship [17]:

(5) $$\alpha =\frac{\sqrt{2}\pi f}{c}\sqrt{\left(\mu^{\prime \prime }\varepsilon^{\prime \prime }-\mu^{\prime }\varepsilon^{\prime }\right)+\sqrt{{\left(\mu^{\prime \prime }\varepsilon^{\prime \prime }-\mu^{\prime }\varepsilon^{\prime }\right)}^{2}+{\left(\mu^{\prime \prime }\varepsilon^{\prime }+\mu^{\prime }\varepsilon^{\prime \prime }\right)}^{2}}}$$

**Figure 6:**
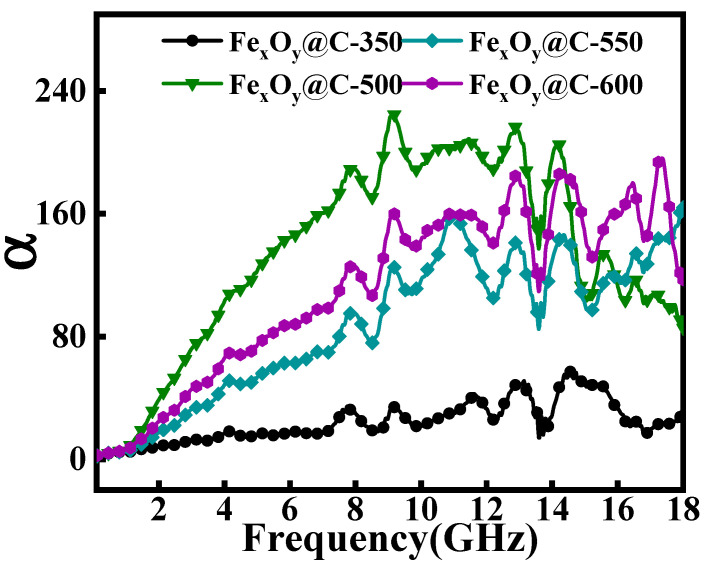
Frequency dependent *α* curves of Fe_x_O_y_@C-T.

Higher α values signify enhanced energy conversion from electromagnetic waves to thermal dissipation through polarization processes and magnetic resonance effects. The frequency dependent *α* curves of Fe_x_O_y_@C-T composites (Figure 6) demonstrates that Fe_x_O_y_@C-500 basically maintains the highest *α* magnitudes, displaying optimal attenuation capability. This exceptional attenuation capability originates from synergistic contributions of enhanced magnetic dissipation and optimized dielectric polarization processes, achieved through controlled magnetic Fe_3_O_4_ phase configuration engineering, an improved electron transition process among Fe^2+^/Fe^3+^ and interfacial charge redistribution at Fe_3_O_4_/C interfaces. The synergistic integration of optimal impedance matching and superior attenuation capacity make Fe_3_O_4_@C composites display improved microwave absorption performance.

**Figure 7:**
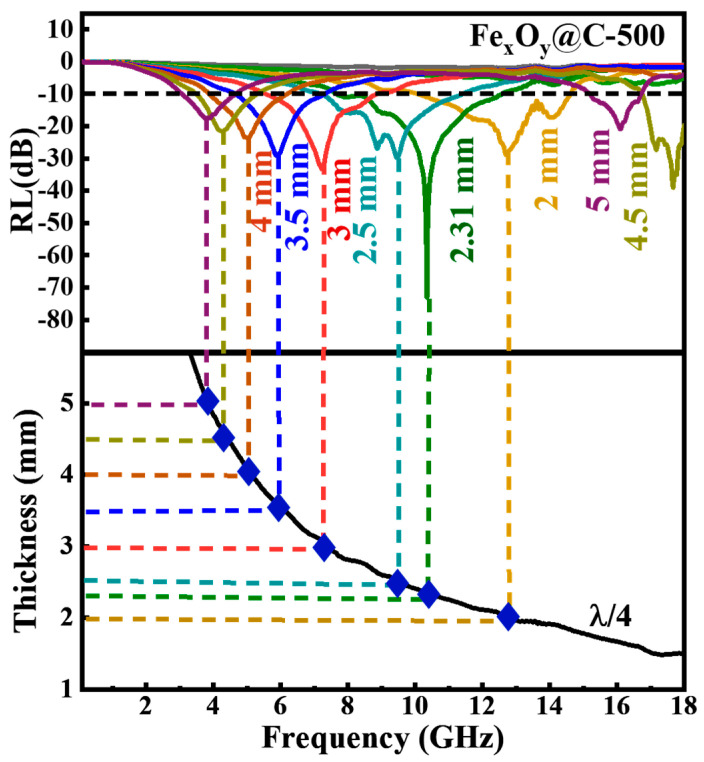
*RL* curves of Fe_x_O_y_@C-500 at different absorber thicknesses and thickness–frequency correlation through *λ*/4 theory.

Microwave dissipation through destructive interference is another mechanism for the consumption of electromagnetic energy, which can be described by the quarter-wavelength matching theory (*λ*/4) [26]:

(6) $$t_{m}=\frac{nc}{4f_{m}\sqrt{\left|\mu_{r}\|\varepsilon_{r}\right|}}\ldots ..(n\;=\;1,\;3,\;5\ldots )$$

where $t_{m}$ is the matching thickness and $f_{m}$ is the corresponding peak frequency. When $t_{m}$ and $f_{m}$ satisfy the *λ*/4 condition, the phase difference between microwave reflections at air–absorber and absorber–metal interfaces reaches 180°, resulting in coherent destructive interference that dissipates propagating waves through anti-phase superposition. The *λ*/4 condition also governs the thickness–frequency correlation of the microwave absorber. The *RL* curves of Fe_x_O_y_@C-500 at different absorber thicknesses (top of Figure 7) clearly reveal the absorption frequency shift to a lower frequency due to increasing the absorber thickness. The lower part of Figure 7 shows the simulated *λ*/4 curves. The blue rhomboid reflects the thickness–frequency correlation obtained from the *RL* curves (top of Figure 7), which is located at the *λ*/4 curves. This indicates that the relationship between matching thickness and frequency of Fe_x_O_y_@C composites matches well with the *λ*/4 theory.

**Figure 8:**
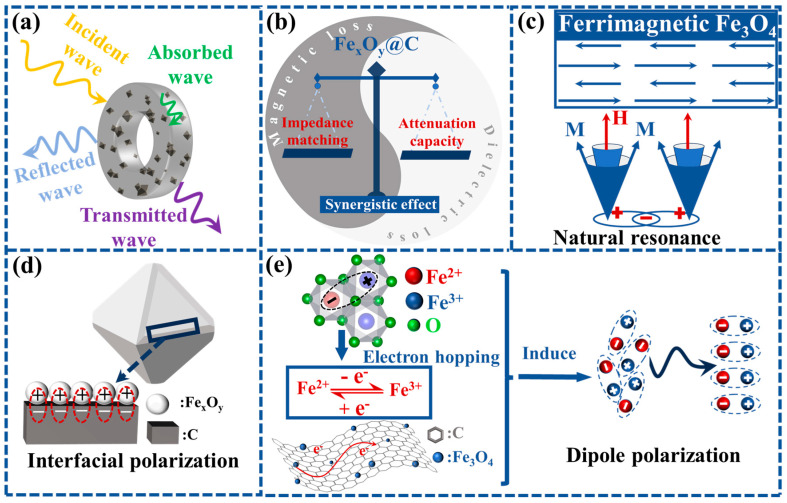
Illustration of microwave absorption mechanisms in Fe_x_O_y_@C composites: (**a**) interactions between microwave absorber and incident electromagnetic waves; (**b**) the synergistic effect of impedance matching and attenuation capacity; (**c**) natural resonance; (**d**) interfacial polarization; (**e**) dipole polarization.

Based on the aforementioned results, Figure 8 schematically illustrates the microwave absorption mechanism of Fe_x_O_y_@C composites enabled by phase and valence state engineering. Upon microwave irradiation, incident waves experience three primary processes: partial surface reflection, partial penetration through the absorber, and energy dissipation via dielectric/magnetic losses (Figure 8a). The combined efforts of phase engineering and valence state modulation give rise to enhanced magnetic loss and optimized dielectric loss, thereby enhancing microwave absorption through two crucial processes. First, impedance matching is improved by balancing the complex permittivity and permeability. This effectively minimizes surface reflection, ensuring that more microwaves enter into the absorber. Second, the microwave attenuation capacity is strengthened. This is achieved through intensified polarization effects and magnetic resonance, which significantly enhance the ability of the absorber to dissipate the microwaves which penetrate it (Figure 8b). Compared to antiferromagnetic Fe_2_O_3_ and paramagnetic FeO, the unpaired electron spins of Fe^2+^/Fe^3+^ in ferrimagnetic Fe_3_O_4_ form a strong net magnetic moment. In an alternating electromagnetic field, the magnetic moment undergoes precession due to natural resonance, and converts electromagnetic energy into heat through interaction with the crystal lattice (relaxation), thereby enhancing magnetic loss. An increase in the ferrimagnetic Fe_3_O_4_ content leads to a strong and broad natural resonance in the GHz frequency range as evidenced in Figure 4e, thereby augmenting magnetic loss (Figure 8c). Due to the conductivity differences between semiconductive Fe_x_O_y_ and conductive carbon, there is localized charge accumulation and separation at the heterogeneous interfaces. Under an alternating electromagnetic field, the redistribution of charges lags behind the change in the electric field direction, leading to relaxation and space charge polarization, which generates interfacial polarization. During this process, charge migration and collisions dissipate electromagnetic energy as heat, resulting in enhanced dielectric loss (Figure 8d). Moreover, the Fe^2+^/Fe^3+^ mixed-valence states promote electron hopping between adjacent Fe^2+^ and Fe^3+^ ions. Crystal defects (such as oxygen vacancies) in Fe_3_O_4_ and the mixed valence states of Fe^2+^/Fe^3+^ lead to uneven charge distribution, forming localized electric dipoles. In an alternating electromagnetic field, dipoles are hindered by lattice constraints or defects and need to overcome potential barriers in order to reorient, causing polarization response to lag behind the electric field change. This accelerates the electron transition process and induces dipole polarization relaxation, resulting in an enhancement of the complex permittivity and dielectric loss (Figure 8e). This synergistic design, which integrates phase modulation, mixed-valence engineering and carbon-confined nanostructures, establishes a robust strategy for enhancing the microwave absorption of MOFs-derived magnetic carbon-based composites by optimizing impedance matching and leveraging dual-loss mechanisms.

## 4. Conclusions

In summary, this work demonstrates a novel strategy for optimizing microwave absorption performance by tailoring phase structures and valence states in MOFs-derived magnetic carbon-based composites. By simply controlling the carbonization temperature, the iron oxide components undergo a temperature-dependent phase evolution (α-Fe_2_O_3_→Fe_3_O_4_→Fe_3_O_4_/FeO), accompanied by tunable valence states of iron ions, leading to tunable magnetic and dielectric losses. The formation of Fe_3_O_4_@C composite emerges as a critical milestone, achieving an *RL*_min_ of −73.14 dB at 10.35 GHz and an effective absorption bandwidth of 4.9 GHz (7.69–12.59 GHz) with a thickness of 2.31 mm. The enhanced microwave absorption performance is attributed to the synergistic effects of improved magnetic loss (via Fe_3_O_4_ phase modulation) and optimized dielectric loss (via polarization relaxation and electron scattering induced by valence state variation and Fe_3_O_4_/C interfaces), resulting in favorable impedance matching and strong microwave attenuation capacity. This study not only provides a feasible strategy for designing advanced microwave absorbers through phase and valence state regulation, but also deepens the understanding of electromagnetic loss mechanisms in composite materials.

## Figures and Tables

**Table 1:** Comparison of the microwave absorption properties of MOFs-derived Fe_3_O_4_@C with other reported Fe_3_O_4_-based composites.

Samples	Thickness (mm)	EAB (GHz)	RL_min_ (dB)	Ref.
Fe_3_O_4_-Fe@CNFs/Al-Fe_3_O_4_-Fe	4.3	5.6	−59.3	[42]
Fe_3_O_4_@spinach-derived carbon	4.5	4.73	−48.81	[43]
CNT film-Fe_3_O_4_-rGO-PDMS	1.42	5.7	−50.5	[44]
3D pleated RGO/MXene/Fe_3_O_4_ microspheres	2.9	4.7	−51.2	[45]
Fe_3_O_4_@walnut shell-derived porous carbon	2.46	2.72	−56.61	[46]
Fe_3_O_4_@C core-shell structure nanospheres	2.9	6.96	−64.89	[47]
Fe_3_O_4_@NPC	3.0	4.5	−65.5	[48]
Fe_3_O_4_@C-T	2.31	4.9	−73.14	This work

## Data Availability

The authors confirm that the data supporting the findings of this study are available within the article.
